# Relationship between TyG-BMI index and glycemic index with diet quality, anthropometric indices, and blood pressure in patients with metabolic syndrome

**DOI:** 10.1097/MD.0000000000041276

**Published:** 2025-01-17

**Authors:** Paria Moulavi, Afsane Ahmadi, Seyed Jalil Masoumi, Morteza Zare, Mahdi Honardoust, Rahil Ranjbar

**Affiliations:** a Student Research Committee, School of Nutrition and Food Sciences, Shiraz University of Medical Sciences, Shiraz, Iran; b Nutrition Research Center, Department of Clinical Nutrition, School of Nutrition and Food Sciences, Shiraz University of Medical Sciences, Shiraz, Iran; c School of Nutrition and Food Sciences, Shiraz University of Medical Science, Shiraz, Iran.

**Keywords:** blood pressure, dietary inflammatory index, glycemic index, metabolic syndrome, TyG-BMI

## Abstract

A recently introduced metric for assessing metabolic syndrome (MetS) is the triglyceride glucose-body mass index (TyG-BMI). Additionally, the glycemic index (GI) is recognized as a significant measure for evaluating conditions associated with blood glucose. In this context, our research explores the correlation between TyG-BMI and GI in relation to diet quality, anthropometric measurements, and blood pressure among individuals diagnosed with MetS. A cross-sectional descriptive-analytical study was conducted on 431 employees with MetS at Shiraz University of Medical Sciences (SUMS). Anthropometric measurements of height, weight, waist circumference (WC), and hip circumference (HC) were taken according to Persian cohort protocols. BMI, C-index, visceral adiposity index (VAI), body adiposity index (BAI), body shape index (ABSI), abdominal volume index (AVI), potential renal acid load (PRAL), TyG-BMI and GI were calculated. A physician measured blood pressure, while the dietary inflammatory index was determined using guidelines. Biochemical parameters were analyzed using standard laboratory techniques. Data analysis was conducted using SPSS software version 21, with a significance threshold set at <.05. A significant correlation was identified between the TyG-BMI index and the PRAL index (β = 0.094, *P*-value = .026), WC (β = 0.627, *P*-value < .001), BAI (β = 0.396, *P*-value < .001), and blood pressure (β = 0.063, *P*-value = .002). Furthermore, the findings indicated a notable association between the GI and blood pressure (β = 0.610, *P*-value < .001). The results of this study suggest that managing the PRAL index, body weight, and blood pressure may be associated with an enhanced status of TyG-BMI. Additionally, appropriate GI may be linked to regulated blood pressure. These findings can inform health-related policy decisions for these patients.

## 1. Introduction

Metabolic syndrome (MetS) constitutes a constellation of metabolic abnormalities that elevate the likelihood of developing cardiovascular disease (CVD), type 2 diabetes, and other health-related issues.^[1]^ The global incidence of MetS is on the rise, making it imperative to identify the factors resulting in both its development and progression.^[2]^ More specifically, an estimated 20 to 25% of the global adult population exhibits a combination of risk factors associated with MetS. In the year 2000, around 32% of adults in the United States were diagnosed with MetS,^[3]^ and in Iran, the prevalence of MetS among adults is 32% overall, with 27% in men and 36% in women.^[4]^

The Triglyceride Glucose-Body Mass Index or TyG-BMI index, a combination of triglyceride and BMI indices, has recently emerged as a valuable tool for evaluating insulin safety and resistance in individuals with MetS.^[5]^ This index offers greater accessibility and cost-effectiveness when compared to more intricate methodologies such as the hyperinsulinemic euglycemic clamp (HIEC) and the Homeostasis Model of Insulin Resistance (HOMA-IR).^[6]^ Investigations in recent literature have highlighted the role of the TyG-BMI index concerning dietary factors in the development of diseases such as MetS, IR, and fatty liver.^[7]^ Furthermore, TyG-BMI index may act as an essential prognostic indicator for individuals at the prediabetic stage. Research findings demonstrate that an increase in the TyG-BMI index is significantly linked to a higher risk of diabetes and suggests the presence of insulin resistance.^[8]^ In other hand, the glycemic index (GI), measuring the impact of food on blood glucose levels, is an essential index for evaluating diseases related to blood sugar.^[9–12]^ Numerous studies have examined the relationship between GI and MetS, with some suggesting a positive association between dietary GI and the prevalence of MetS.^[13–15]^

Recent research has identified innovative anthropometric indices, including a body shape index (ABSI) and abdominal volume index (AVI), which demonstrate enhanced effectiveness in detecting MetS among non-overweight individuals compared to traditional metrics.^[16]^ Dietary factors also play a significant role in the development and progression of MetS including the dietary inflammatory index (DII) which was established as a quantitative instrument designed to evaluate the impact of dietary patterns on health outcomes and chronic diseases.^[17,18]^ Furthermore, recently, several studies have investigated the role of diet-related metabolic acidosis in the pathogenesis of diseases such as MetS, diabetes, and CVD. In this context, A multitude of studies have documented the correlation between metabolic risk factors and dietary acid load (DAL), which can be quantified using measures such as potential renal acid load (PRAL), net endogenous acid production (NEAP), or both.^[19]^ Eventually, due to limited investigations in this area, we aimed to find the relationship between the TyG-BMI and GI with diet quality, anthropometric indices, and blood pressure in patients with MetS.

## 2. Materials and Methods

### 2.1. Study design and setting

This study is a cohort-based, cross-sectional, descriptive-analytical study; conducted in the Shiraz University Medical Science (SUMS) during 2017 to 2020 and monitored by the PERSIAN cohort central committee; to ensure authentic data collection based on the PERSIAN protocol.

Shiraz University of Medical Sciences Employees Health Cohort Study (SUMS EHCS), a branch of the Persian cohort, is a prospective study aiming to include 10,000 employees aged 20 to 70 years from Shiraz, Fars, Iran. While the Ministry of Health and Medical Education (MOHME) oversaw the project, investigators at SUMS performed it. The study received approval from SUMS ethical committee with the approval code IR.SUMS.SCHEANUT.REC.1401.071. Before taking part in the research, all participants signed a written informed consent form.

### 2.2. Selection of participant

**Figure 1.**
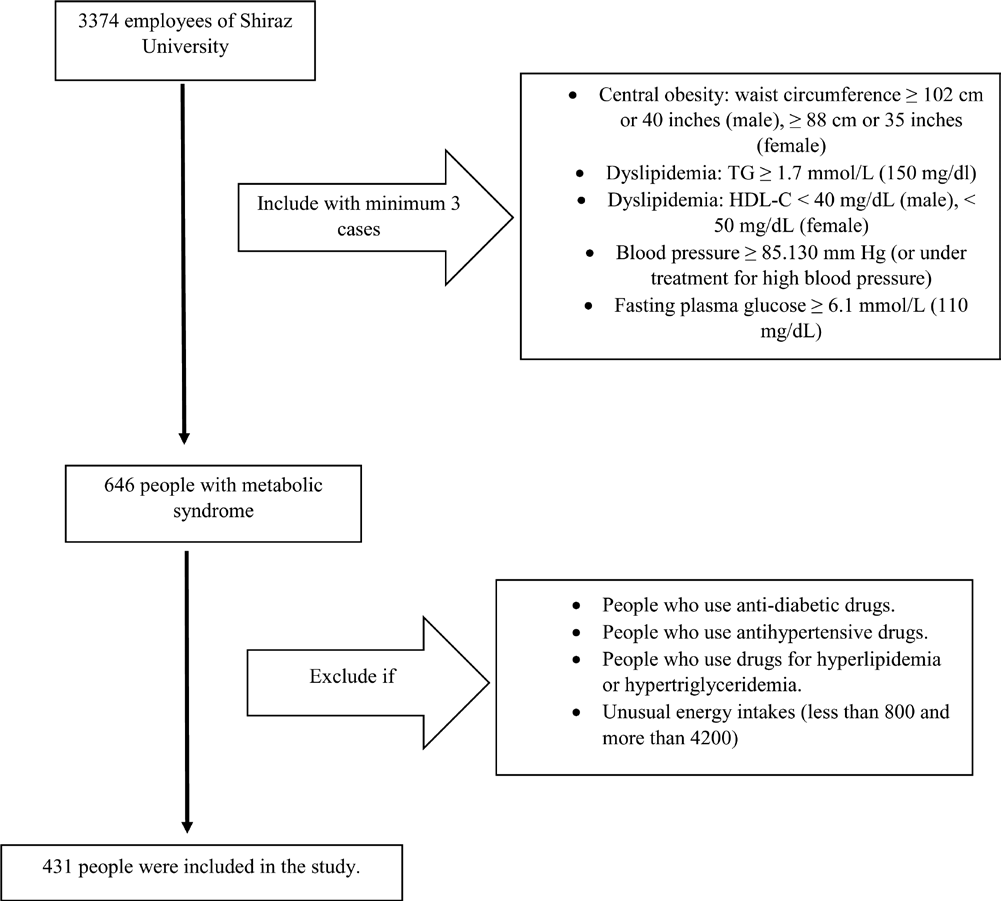
Flow graph of individual recruitment.

The research methodology utilized in this study incorporates a census sampling technique. Potential participants included patients with MetS, aged 20 to 70 years who did not meet the exclusion criteria of recent history or current use of antidiabetic drugs, antihypertensive drugs, drugs for hyperlipidemia or hypertriglyceridemia, unusual energy intakes (<800 and more than 4200). The flow graph illustrating individual recruitment is presented in Figure 1.

### 2.3. Data collection and measurement

The demographic, biochemical, physical activity, anthropometric and dietary data required for the study were collected from the SUMS EHCS. Several study variables, including ABSI, visceral adiposity index (VAI), AVI, body adiposity index (BAI), C-Index, DII, PRAL, DAL, NEAP, TyG-BMI, and GI, were calculated through the formulas.

#### 2.3.1. Blood pressure measurement

A general practitioner used a standard sphygmomanometer to measure blood pressure twice on the right arm and twice on the left arm. The final number reported was the average of the recorded numbers.

#### 2.3.2. Evaluation of biochemical indicators

Participants were required to fast for at least 12 hours before the test. Ten ml of blood was drawn from each person to measure fasting blood sugar level and total cholesterol (Chol) concentration, high-density lipoprotein cholesterol (HDL-Chol), low-density lipoprotein cholesterol (LDL-Chol), and triglycerides (TG).

#### 2.3.3. Evaluation of physical activity

The international physical activity questionnaire was enrolled for gathering data on participants physical activity levels, categorized them into light, moderate and intense based on metabolic equivalent of task (Met) per week as Vasheghani-Farahani et al^[20]^ described.

#### 2.3.4. Food frequency questionnaire (FFQ)

Trained experts administered a 121-item FFQ questionnaire in person.^[21]^ The questionnaire included information on the amount and frequency of food consumption in detail on a daily, weekly, monthly, and yearly basis over the past year. The obtained information was entered into Nutritionist IV software to evaluate participants’ intake, and SPSS version 21 software was used to assess the data.

#### 2.3.5. Anthropometric measurements

Height was measured in a standing position without shoes using tape while the shoulders were in their natural position. Weight was measured with minimal clothing and without shoes using a digital scale. BMI was calculated for each participant based on dividing weight (in kilograms) by the square of height (in meters). Waist circumference (WC) was measured using an inflexible tape in the narrowest part of light clothing, and hip circumference (HC) was measured in the widest part of the hip without any pressure on the body surface.^[22]^

#### 2.3.6. Assessment of GI

Using the FFQ questionnaire, daily carbohydrate consumption was calculated for all participants, and the carbohydrates of specified foods were used to determine the GI of each person’s diet.^[23]^

#### 2.3.7. Acid load measurement

Acid load was measured using 3 indicators. The PRAL index; determines the amount of intestinal absorption of 5 nutrients, including protein, potassium, calcium, phosphorus, and magnesium.^[24]^ The NEAP index determines the ratio of protein to potassium in food.^[25]^ The DAL index includes the 5 nutrients mentioned above and the body surface.^[26]^

#### 2.3.8. Evaluation of DII

To evaluate the DII, nutritional information was collected through the FFQ questionnaire. The DII questionnaire includes 45 food parameters, including macronutrients, micronutrients (vitamins and minerals), and flavonoids. The scoring method is based on comparing the daily intake of individuals with the global average and the inflammatory factor of each parameter. Scores range from +1 to −1, with 1 indicating anti-inflammatory properties, +1 indicating pro-inflammatory properties, and 0 indicating no effect on the inflammation process. A score of −8.87 is reported as the most anti-inflammatory, and a score of +7.98 is reported as the most pro-inflammatory food item. To calculate the food inflammatory index, the intake of food parameters is subtracted from the global average intake and then divided by the global standard deviation to obtain the Z-score. The *Z*-score values are converted into percentiles, multiplied by 2, and subtracted by 1. Scores are multiplied by the overall inflammatory score, and the values are added to calculate the DII’s final score.^[27]^

#### 2.3.9. MetS definition

MetS diagnosis was determined using the NCEP ATPIII criteria.^[28]^

#### 2.3.10. Calculation

**TyG-BMI** = Ln [FBG (mg/dL) × fasting TG (mg/dL)/2] * BMI

**Glycemic index** = (GI of food * amount of available carbohydrates)/ amount of total available carbohydrates

**ABSI** = WC/BMI^2/3^ × height^1/2^

**VAI** = (WC (cm)/(39.68 + (1.88*BMI)))*(TG/1.03)*(1.31/HDL) for males and VAI = (WC(cm)/ (36.58 + (1.89*BMI)))*(TG/0.81)*(1.52/HDL) for females.

**AVI **= [(2*WC^2^) + 0.7 (WC − HC)^2^]/ 1000

**C-Index** = WC (m)/ (0.109*[Wt (kg)/ Ht (m)]^-1/2^)

**BAI **= [HC (cm)/H (m)^1.5^] − 18

**PRAL** = 0.49 × protein (g/d) + 0.037 × phosphorus (mg/d) − 0.021 × potassium (mg/d) − 0.026 magnesium (mg/d) − 0.013 × calcium (mg/d).

**NEAP** = 54.5 × [protein intake (g/d)/potassium intake (mEq/d)] − 10.2

**DAL** = PRAL + (body surface area [m^2^] × 41[mEq/d]/1.73 m^2^)

### 2.4. Data analysis

The data were entered into a file and analyzed using SPSS version 21. Descriptive statistics were used to present demographic data, including frequencies, means with standard deviations, and medians with interquartile ranges. Univariate linear regression models were used to determine the relation between TyG-BMI and GI with anthropometric indices, DII, dietary acidity, and blood pressure. According to the results of multiple regression analysis, after adjusting confounding factors, variables with a significance level of <.2 were entered into a multivariate regression model. The significance level for 2-sided tests was set at .05. The relationship of each variable was first assessed through univariate analysis. To address collinearity among the variables, the VIF index was utilized.

## 3. Results

During the study, 3374 patients were screened, and 646 patients with MetS were enrolled. Of those, 215 were excluded based on the inclusion criteria, while 431 patients were included in the study. Of the participants, 207 were male (48%) and 224 were female (52%). In terms of educational level, the highest percentage of participants had a bachelor’s degree at 41.1%, followed by a diploma at 20.9%, master’s degree at 14.3%, post-diploma at 13.9%, elementary school at 4.6%, guidance school at 3.2%, doctoral degree at 1.5%, and illiterate at 0.5% (Table 1).

**Table 1:** Baseline characteristics of study subjects.

Variables	Mean	Median	Standard. deviation	IQR
FBS	99.49	20.70	97.13	18
TG	206.51	94.28	185.91	87
SBP	114.59	14.99	114	22
DBP	76.74	10.85	78	15
BMI	29.13	3.84	28.95	4.68
WC	100.6	99.5	9.56	12
HC	106.04	105.2	7.27	9
Age	45.11	41	65.64	10
AVI	20.47	19.85	4.02	4.86
BAI	31.43	30.99	4.67	6.15
PRAL Index	323.74	306.16	107.59	138.31
Dal Index	324.72	307.22	107.62	138.1
NEAP Index	36.29	33.34	21.76	16.08
Dietary inflammatory index	0.083	0.08	2.067	3.39
C-Index	1.32	1.32	0.06	0.08
ABSI	0.08	0.08	0.004	0.01
TyG-BMI Index	143.1	141.62	19.99	23.63
Glycemic Index	56.75	56.29	6.006	8.57
Blood pressure	2.11	2	1.18	2

Data were presented in the form of mean ± SD, median (IQR), or counts (percentages), depending on its type.

Abbreviations: ABSI = a body shape index, AVI = abdominal volume index, BAI = body adiposity index, BMI = body mass index, BRI = body roundness index, CI = conicity, DBP = diastolic blood pressure, FPG = fasting plasma glucose, HC = hip circumference, HDL = high-density lipoprotein cholesterol, LDL = low-density lipoprotein cholesterol, PRAL = potential renal acid load, SBP = systolic blood pressure, TG = triglycerides, TyG-BMI = triglyceride glucose-body mass index, VAI = visceral adiposity index, WC = waist circumference.

### 3.1. Factors associated with the TyG-BMI index were examined using single and multiple linear regression

The analysis of gender differences revealed that men exhibited a lower TyG-BMI index compared to women (β = −0.342, *P*-value < .001). Additionally, individuals with nonacademic education demonstrated a reduced TyG-BMI index (β = −0.260, *P*-value = .043). Furthermore, an increase in the PRAL index was associated with a rise in the TyG-BMI index (β = 0.094, *P*-value = .026), suggesting that the PRAL index significantly influenced the TyG-BMI index (Table 2).

**Table 2:** Prediction of demographic and dietary factors affecting the TyG-BMI index was conducted using single and multiple variable linear regression models in the cohort of employees of Shiraz University of Medical Sciences.

Univariate regression					Multivariate regression			
Independent variables	Regression coefficient (β)	Significance level	95% CI		Regression coefficient (β)	Significance level	Confidence interval	
			lower	upper				
Gender	Reference Women: −0.391	<0.001	−0.577	−0.205	−0.364	<0.001	−0.551	0.178
Education	Reference Academic: −0.254	0.053	−0.551	0.003	−0.260	0.043	−0.511	−0.009
Age	−0.002	0.038	0.000	0.003	0.001	0.049	0.517	3.695
PRAL Q1 to Q4	0.108	0.012	−0.024	0.192	0.094	0.026	0.011	0.176
DII Q1 to Q4	−0.034	0.426	−0.119	0.050	–	–	–	

*P*-value > .05 was considered significant.

In order to eliminate the confounding effect, a multivariate linear regression model (with the enter method) was used.

The present model adjusted for gender, age, education, PRAL index, and dietary inflammatory index (DII).

PRAL index: first quartile: ≤ 244.3143, second quartile: 244.3144 to 306.1659, third quartile: 306.166 to 382.6225, fourth quartile: ≥382.6226.

Food inflammation index: first quartile: ≤−1.6000, second quartile: −1.6001 to 0.0800, third quartile: 0.0801 to 1.7900, fourth quartile: ≥1.7901.

Age: first quartile: ≤37, second quartile: 37 to 41, third quartile: 41 to 47, fourth quartile: ≥47.

Abbreviations: PRAL = potential renal acid load, TyG-BMI = triglyceride glucose-body mass index.

Moreover, an increase in WC corresponded with a higher TyG-BMI index (β = 0.627, *P*-value < .001), and similarly, an increase in BAI was linked to a greater TyG-BMI index (β = 0.396, *P*-value < .001). In addition, elevated blood pressure levels also contributed to an increase in the TyG-BMI index (β = 0.063, *P*-value = .002) (Table 3).

**Table 3:** Prediction of anthropometric and cardiometabolic factors affecting the TyG-BMI index was conducted using single and multiple variable linear regression models in the cohort of employees of Shiraz University of Medical Sciences.

Univariate regression					Multivariate regression			
Independent variables	Regression coefficient (β)	Significance level	95% CI		Regression coefficient (β)	Significance level	Confidence interval	
			lower	upper				
Gender	Reference Women: −0.391	<0.001	−0.577	−0.205	−0.342	<0.001	0.001	0.000
Education	Reference Academic: −0.254	0.053	−0.551	0.003	–	–	–	
Age	−0.002	0.038	0.000	0.003	–	–	–	
WC Q1 to Q4	0.613	<0.001	0.551	0.675	0.627	<0.001	0.541	0.713
HC Q1 to Q4	0.574	<0.001	0.509	0.640	–	–	–	
Blood pressure Normal - type 2 hypertension	−0.034	0.426	−0.119	0.050	0.063	0.002	0.025	0.008
BAI Q1 to Q4	0.33	<0.001	0.251	0.408	0.396	<0.001	0.321	0.470

*P*-value > .05 was considered significant.

In order to eliminate the confounding effect, a multivariate linear regression model (with the enter method) was used.

The present model adjusted for gender, age, education, waist circumference, hip circumference, body fat index, and blood pressure.

Abdominal circumference: first quartile: ≤93.6/, second quartile: 93.6 to 99.5, third quartile: 93.5 to 106, fourth quartile: ≥106.

Waist circumference: first quartile: ≤32/81, second quartile: 81/113 to 33/77, third quartile: 113/162 to 78/97, fourth quartile: ≥162/98.

Age: first quartile: ≤37, second quartile: 37 to 41, third quartile: 41 to 47, fourth quartile: ≥47.

BAI: first quartile: ≤28.1579, second quartile: 28.158 to 30.9927, third quartile: 30.9928 to 34.3126, fourth quartile: ≥34.3127.

Blood pressure: It was divided into 4 categories: normal, elevated, type 1 blood pressure, and type 2 blood pressure.

Abbreviations: BAI = body adiposity index, HC = hip circumference, TyG-BMI = triglyceride glucose-body mass index, WC = waist circumference.

### 3.2. Factors associated with the GI were examined using single and multiple linear regression

Based on gender evaluation, males had a greater GI compared to females (β = 0.251, *P*-value = .038). Furthermore, a rise in HC correlated with a higher GI values (β = 0.106, *P*-value = .041), and elevated blood pressure was also associated with an increase in the GI (β = 0.610, *P*-value < .001) (Table 4).

**Table 4:** Prediction of demographic and dietary factors affecting the glycemic index was carried out using single and multiple variable linear regression models in the cohort of employees of Shiraz University of Medical Sciences.

Univariate regression					Multivariate regression			
Independent variables	Regression coefficient (β)	Significance level	95% CI		Regression coefficient (β)	Significance level	95% CI	
			Lower	Upper			Lower	Upper
Gender	Reference Women: −0.493	<0.001	−0.678	−0.309	0.251	0.038	0.014	0.487
Education	Reference Academic: 0.018	0.890	−0.240	0.277	–	–	–	
Age Q1 to Q4	0.000	0.597	−0.002	0.001	–	–	–	
Physical activity (MetS/wk)	0.21	0.520	1.00	1.03	–	–	–	
WC Q1 to Q4	0.92	0.023	0.007	0.177	–	–	–	
HC Q1 to Q4	0.110	0.011	0.025	0.195	0.106	0.041	0.004	0.208
Blood pressure Normal-type 2 hypertension	0.563	<0.001	0.524	0.623	0.61	<0.001	0.54	0.679
BAI Q1 to Q4	0.33	<0.001	0.251	0.408	–	–	–	
C-Index Q1 to Q4	−0.075	0.082	−0.160	0.010	–	–	–	
ABSI Q1 to Q4	−0.103	0.017	−0.187	−0.018	–	–	–	
VAI Q1 to Q4	−0.083	0.054	−0.168	0.001	–	–	–	
PRAL Q1 to Q4	0.045	0.295	−0.039	0.13	–	–	–	
NEAP Q1 to Q4	0.47	0.272	−0.037	0.132	–	–	–	
DII Q1 to Q4	−0.028	0.52	−0.113	0.057	–	–	–	

*P*-value > .05 was considered significant.

In order to eliminate the confounding effect, a multivariate linear regression model (with the enter method) was used.

The current model adjusted for gender, age, waist circumference, hip circumference, body shape index, density index, visceral fat index, body fat index, abdominal volume index, PRAL, NEAP index, dietary inflammatory index, and blood pressure.

Abdominal circumference: first quartile: ≤93.6/, second quartile: 93.6 to 99.5, third quartile: 93.5 to 106, fourth quartile: ≥106.

Waist circumference: first quartile: ≤32/81, second quartile: 81/113 to 33/77, third quartile: 113/162 to 78/97, fourth quartile: ≥162/98.

Age: first quartile: ≤37, second quartile: 37 to 41, third quartile: 41 to 47, fourth quartile: ≥47.

C-Index: first quartile: ≤1.2860, second quartile: 1.2861 to 1.3269, third quartile: 1.327 to 1.3704, fourth quartile: ≥1.3705.

ABSI: first quartile: ≤0.08, second quartile: 0.0825 to 0.0801, third quartile: 0.0826 to 0.0853, fourth quartile: ≥0.0854.

PRAL index: first quartile: ≤ 244.3143, second quartile: 244.3144 to 306.1659, third quartile: 306.166 to 382.6225, fourth quartile: ≥382.6226.

NEAP index: first quartile: ≤25.8443, second quartile: 25.8444–33.3486, third quartile: 33.3487–41.9263, fourth quartile: ≥41.9264.

VAI: first quartile: ≤5.7479, second quartile: 5.748 to 7.4221, third quartile: 7.4222 to 9.7367, fourth quartile: ≥9.7368.

BAI: first quartile: ≤28.1579, second quartile: 28.158 to 30.9927, third quartile: 30.9928 to 34.3126, fourth quartile: ≥34.3127.

Food inflammation index: first quartile: ≤−1.6000, second quartile: −1.6001 to 0.0800, third quartile: 0.0801 to 1.7900, fourth quartile: ≥1.7901.

Blood pressure: It was divided into 4 categories: normal, elevated, type 1 blood pressure, and type 2 blood pressure.

Abbreviations: ABSI = a body shape index, BAI = body adiposity index, DII = dietary inflammatory index, HC = hip circumference, NEAP = net endogenous acid production, PRAL = potential renal acid load, VAI = visceral adiposity index, WC = waist circumference.

## 4. Discussion

Since MetS is not classified as a singular disease; rather, it encompasses a collection of risk factors associated with CVD, it is crucial to evaluate different aspects affecting it. In this regard, the findings of our study offer valuable insights into the elements that contribute to MetS. Our research revealed that the TyG-BMI index was directly linked to PRAL index, WC, BAI and also blood pressure status. In addition, GI was positively correlated with HC and blood pressure levels.

Our findings showed that there is an inverse association between gender and the TyG-BMI index, with men showing lower TyG-BMI index values compared to women. These gender-specific outcomes may be attributed to variations in lean and body fat distribution. Also, participants with nonacademic education had lower TyG-BMI index. Although some studies reported lower levels of education associated with BMI elevation,^[29,30]^ we indicated different findings. Another interesting findings in this study was a direct correlation between the TyG-BMI, PRAL and some anthropometric and body composition indices, including WC and BAI.

While research on the correlation between PRAL and TyG-BMI is limited, a meta-analysis indicated that a high DAL is linked to increased serum triglyceride levels and a higher prevalence of obesity.^[19]^ Also, the research conducted by Rezazadegan et al in 2022 indicated that DAL, measured by PRAL and NEAP, could be linked to an increased likelihood of developing an unhealthy phenotype in overweight or obese adolescents. Consequently, reducing the intake of acidic foods while enhancing the consumption of fruits and vegetables may positively influence the metabolic health of adolescents and help avert the onset of MetS. This study also determined that a diet characterized by a lower DAL can be advantageous for IR and beta-cell function.^[31]^ It has been proposed that the accumulation of hydrogen ions from acidogenic diets contributes to weight gain and obesity.^[32]^ Also, an increased DAL may be associated with obesity, diabetes, and lipid disorders, primarily due to its influence on enhancing cortisol secretion. Furthermore, it has demonstrated that imbalances in acid and base levels can lead to IR and a reduction in insulin secretion.^[33]^

The rise in the TyG-BMI index among individuals exhibiting elevated WC and body fat percentage can be linked to the substantial amounts of fat found in adipose tissue, particularly in the abdominal region. This fat accumulation serves as an independent contributor to the onset of IR and the metabolic alterations that are associated with risks for CVDs and diabetes.^[34,35]^ Our findings are somewhat similar to a recent cohort study among middle-aged and older individuals evaluating various obesity- and lipid-related metrics in predicting MetS. In males, the TyG-BMI emerged as the most reliable indicator for identifying MetS.^[36]^ Another study by Mijangos-Trejo and colleagues demonstrated that the TyG-BMI and TyG-WC metrics enhanced predictive accuracy in identifying liver steatosis.^[37]^ Also, Khamseh et al introduce TyG-BMI and TyG-WC indices as straightforward, practical, and cost-effective instruments for the screening of nonalcoholic fatty liver disease (NAFLD) and liver fibrosis in clinical environments.^[38]^ Numerous studies indicate that NAFLD is associated with various conditions, including type 2 diabetes, abdominal obesity, IR, dyslipidemia, hypertension, and CVDs. Based on Li et al findings, in comparison to the TyG index, TG/HDL-C, and HOMA-IR, the TyG-BMI has proven to be a more effective predictor of NAFLD in individuals with type 2 diabetes.^[39]^ There is also another evidence that TyG-BMI is the most effective predictor of IR.^[40]^ IR contributes to the emergence of an atherogenic dyslipidemic profile, along with prothrombotic and pro-inflammatory conditions. In individuals with IR, dyslipidemia and body fat accumulation is also remarkable.^[41]^

Another finding of the current study indicated a direct association between the TyG-BMI index and blood pressure. However, research regarding the relationship between the dynamic fluctuations of TyG-BMI and the risk of hypertension is rare. Theoretically, IR is a defining feature of various conditions, notably the MetS. Although the involvement of IR in the development of hyperglycemia and dyslipidemia has been extensively researched, there is comparatively less understanding regarding its contribution to the hypertension. IR may be perceived as a significant contributor to the development of hypertension through multiple pathways including enhanced sodium reabsorption in renal tubules, stimulation of the sympathetic nervous system, and modifications in vascular resistance due to elevated calcium levels in smooth muscle cells.^[42]^ In this regard, a study by Peng et al, revealed that the TyG-BMI has a notable positive correlation with both normal-high blood pressure and hypertension. Furthermore, TyG-BMI proved to be more effective in identifying when compared to the BMI and TyG index individually.^[43]^ Similarly, a linear correlation was identified between cumulative TyG-BMI and the likelihood of developing hypertension in 2 different studies.^[44,45]^ Although, TyG-BMI was highly correlated with HTN in normalglycemia in Huang et al study, further experiments involving diverse populations are required in future.^[46]^

Our research has suggested that male gender was associated with an increase in the GI. In this regard, in Burger et al study, GI, along with elevated consumption of carbohydrates and starch, were linked to a greater risk of CVD among men compare to women.^[47]^ Also, there was a direct relationship between GI with HC and blood pressure in current research. A prevailing theory suggests that a diet characterized by a high GI may elevate the risk of weight gain. HC, while directly related to WC and BMI, has been demonstrated to have an inverse relationship with blood glucose levels, blood pressure, and lipid profiles. Furthermore, it is linked to a decreased risk of CVDs, as well as lower all-cause and cardiovascular mortality rates, especially when accounting for WC.^[48]^ It should be noted that BMI, WC, and waist/hip ratio, not HC, have been shown to be correlated with MetS and type 2 diabetes.^[49]^ Its suggested that WC, WHR, and WHtR are better indicators of obesity which can demonstrate superior predictive capabilities for poor glycemic control.^[50]^ Also, a study showed that high GI diets can contribute to an increase in body weight, body fat percentage, and WC among women, particularly those who are sedentary.^[51]^

In line with the direct correlation between GI and blood pressure there are evidences emphasized on attribution of the high GI diet and induced hyperinsulinemia and IR through increased blood glucose levels. These physiological conditions are associated with enhanced carbohydrate oxidation, reduced fat oxidation, and possibly an increased accumulation of energy in adipose tissue. The pronounced insulin response can result in significant fluctuations in blood glucose levels, which may trigger a return of hunger sooner than expected, thereby prompting an earlier onset of the subsequent meal.^[51]^ Hyperinsulinemia is believed to stimulate increased activity of the sympathetic nervous system, which in turn elevates heart rate, cardiac output, vascular resistance, and sodium retention, ultimately contributing to an increase in blood pressure.^[52]^ In this context, a systematic review and meta-analysis of healthy individuals suggested that adhering to a diet with a lower GI could result in significant decreases in blood pressure.^[53]^ Another study showed that excessive consumption of carbohydrates, particularly from foods with a high GI, may negatively affect blood pressure, especially in females.^[23]^ However, in a clinical trial investigation, the low GI diet did not result in any changes to weight or blood pressure after a 10-week intervention when compared to the healthy nutritional recommendation diet.^[54]^

The limitations of the present study includes the lack of access to several biochemical inflammatory indicators, the cross-sectional nature of the study, the participants’ lifestyle modifications and changes over time due to their high level of education, and the unavailability of all 45 items of the food inflammatory index. It is noted that these factors may have influenced the results.

## 5. Conclusion

To sum up, the findings of this research indicate that the management of the diet quality, body composition, and blood pressure may correlate with an improved status of TyG-BMI. Furthermore, proper GI may be associated with controlled blood pressure status. These insights could guide health-related policy decisions in this area.

## Acknowledgments

The authors express their gratitude to the participants of the cohort of Persian Cohort Center of Shiraz University employees for their valuable contributions. The present study was extracted from a M.S. thesis done by PM.

## Author contributions

**Conceptualization:** Paria Moulavi, Afsane Ahmadi, Seyed Jalil Masoumi, Mahdi Honardoust, Rahil Ranjbar.

**Data curation:** Paria Moulavi, Afsane Ahmadi, Seyed Jalil Masoumi.

**Formal analysis:** Afsane Ahmadi, Morteza Zare, Paria Moulavi.

**Funding acquisition:** Afsane Ahmadi.

**Investigation:** Afsane Ahmadi, Paria Moulavi.

**Methodology:** Afsane Ahmadi, Paria Moulavi, Morteza Zare, Seyed Jalil Masoumi.

**Project administration:** Afsane Ahmadi, Seyed Jalil Masoumi.

**Resources:** Seyed Jalil Masoumi, Afsane Ahmadi.

**Software:** Afsane Ahmadi, Paria Moulavi, Morteza Zare.

**Supervision:** Afsane Ahmadi.

**Validation:** Afsane Ahmadi, Morteza Zare, Seyed Jalil Masoumi.

**Visualization:** Afsane Ahmadi, Paria Moulavi, Mahdi Honardoust, Rahil Ranjbar.

**Writing – original draft:** Afsane Ahmadi, Paria Moulavi, Mahdi Honardoust, Rahil Ranjbar.

**Writing – review & editing:** Afsane Ahmadi, Paria Moulavi, Mahdi Honardoust.

## References

[1] Fahed G Aoun L Bou Zerdan M. Metabolic syndrome: updates on pathophysiology and management in 2021. Int J Mol Sci. 2022;23:786. 35054972 10.3390/ijms23020786 PMC8775991

[2] Engin A. The definition and prevalence of obesity and metabolic syndrome. Adv Exp Med Biol. 2017;960:1–17. 28585193 10.1007/978-3-319-48382-5_1

[3] Awuchi CG Echeta CK Igwe VS. Diabetes and the nutrition and diets for its prevention and treatment: a systematic review and dietetic perspective. Health Sci Res. 2020;6:5–19.

[4] Dalvand S Bakhshi E Zarei M Asl MT Gheshlagh RG. Prevalence of Metabolic Syndrome in Iran: a systematic review and meta-analysis. Med Surg Nurs J. 2017;5:e67928.

[5] Wang X Liu J Cheng Z Zhong Y Chen X Song W. Triglyceride glucose-body mass index and the risk of diabetes: a general population-based cohort study. Lipids Health Dis. 2021;20:1–10. 34488806 10.1186/s12944-021-01532-7 PMC8420033

[6] Pacini G Mari A. Methods for clinical assessment of insulin sensitivity and β-cell function. Best Pract Res Clin Endocrinol Metab. 2003;17:305–22. 12962688 10.1016/s1521-690x(03)00042-3

[7] McCracken E Monaghan M Sreenivasan S. Pathophysiology of the metabolic syndrome. Clin Dermatol. 2018;36:14–20. 29241747 10.1016/j.clindermatol.2017.09.004

[8] Pryzbek M Liu J. Association between upper leg length and metabolic syndrome among US elderly participants-results from the NHANES (2009-2010). J Geriatr Cardiol. 2016;13:58–63. 26918014 10.11909/j.issn.1671-5411.2016.01.017 PMC4753013

[9] Jenkins DJ Dehghan M Mente A. Glycemic index, glycemic load, and cardiovascular disease and mortality. N Engl J Med. 2021;384:1312–22. 33626252 10.1056/NEJMoa2007123

[10] Rahimlou M Morshedzadeh N Karimi S Jafarirad S. Association between dietary glycemic index and glycemic load with depression: a systematic review. Eur J Nutr. 2018;57:2333–40. 29744611 10.1007/s00394-018-1710-5

[11] Vlachos D Malisova S Lindberg FA Karaniki G. Glycemic Index (GI) or Glycemic Load (GL) and dietary interventions for optimizing postprandial hyperglycemia in patients with T2 diabetes: a review. Nutrients. 2020;12:1561. 32471238 10.3390/nu12061561 PMC7352659

[12] Vega-López S Venn BJ Slavin JL. Relevance of the glycemic index and glycemic load for body weight, diabetes, and cardiovascular disease. Nutrients. 2018;10:1361. 30249012 10.3390/nu10101361 PMC6213615

[13] Zhang J-Y Jiang Y-T Liu Y-S Chang Q Zhao Y-H Wu Q-J. The association between glycemic index, glycemic load, and metabolic syndrome: a systematic review and dose–response meta-analysis of observational studies. Eur J Nutr. 2020;59:451–63. 31680212 10.1007/s00394-019-02124-z

[14] Juanola-Falgarona M Salas-Salvadó J Buil-Cosiales P.; PREvencion con DIeta MEDiterranea Study Investigators. Dietary glycemic index and glycemic load are positively associated with risk of developing metabolic syndrome in middle-aged and elderly adults. J Am Geriatr Soc. 2015;63:1991–2000. 26480969 10.1111/jgs.13668

[15] Finley CE Barlow CE Halton TL Haskell WL. Glycemic index, glycemic load, and prevalence of the metabolic syndrome in the cooper center longitudinal study. J Am Diet Assoc. 2010;110:1820–9. 21111092 10.1016/j.jada.2010.09.016

[16] Wu L Zhu W Qiao Q Huang L Li Y Chen L. Novel and traditional anthropometric indices for identifying metabolic syndrome in non-overweight/obese adults. Nutr Metab (Lond). 2021;18:3. 33407674 10.1186/s12986-020-00536-x PMC7788902

[17] Zuydam NV Wielscher M McCarthy M Jarvelin M-R. Increased obesity is causal for increased inflammation—a Mendelian randomisation study. Diabetes. 2018;67(Supplement_1):LB59.

[18] Carvalho CA Silva AAM Assunção MCF. The dietary inflammatory index and insulin resistance or metabolic syndrome in young adults. Nutrition. 2019;58:187–93. 30504010 10.1016/j.nut.2018.07.014

[19] Abbasalizad Farhangi M Nikniaz L Nikniaz Z. Higher dietary acid load potentially increases serum triglyceride and obesity prevalence in adults: an updated systematic review and meta-analysis. PLoS One. 2019;14:e0216547. 31071141 10.1371/journal.pone.0216547 PMC6508739

[20] Vasheghani-Farahani A Tahmasbi M Asheri H Ashraf H Nedjat S Kordi R. The Persian, last 7-day, long form of the International Physical Activity Questionnaire: translation and validation study. Asian J Sports Med. 2011;2:106–16. 22375226 10.5812/asjsm.34781 PMC3289200

[21] Eghtesad S Hekmatdoost A Faramarzi E. Validity and reproducibility of a food frequency questionnaire assessing food group intake in the PERSIAN cohort study. Front Nutr. 2023;10:1059870. 37599697 10.3389/fnut.2023.1059870 PMC10436288

[22] Jamshidi S Masoumi SJ Abiri B Vafa M. The effects of synbiotic and/or vitamin D supplementation on gut-muscle axis in overweight and obese women: a study protocol for a double-blind, randomized, placebo-controlled trial. Trials. 2022;23:631. 35927757 10.1186/s13063-022-06598-x PMC9351060

[23] Gopinath B Flood VM Rochtchina E Baur LA Smith W Mitchell P. Influence of high glycemic index and glycemic load diets on blood pressure during adolescence. Hypertension. 2012;59:1272–7. 22493075 10.1161/HYPERTENSIONAHA.112.190991

[24] Bahadoran Z Mirmiran P Khosravi H Azizi F. Associations between dietary acid-base load and cardiometabolic risk factors in adults: the Tehran Lipid and Glucose Study. Endocrinol Metab (Seoul). 2015;30:201–7. 25433661 10.3803/EnM.2015.30.2.201 PMC4508265

[25] Parmenter BH Dymock M Banerjee T Sebastian A Slater GJ Frassetto LA. Performance of predictive equations and biochemical measures quantifying net endogenous acid production and the potential renal acid load. Kidney Int Rep. 2020;5:1738–45. 33102966 10.1016/j.ekir.2020.07.026 PMC7569692

[26] Storz MA Ronco AL Hannibal L. Observational and clinical evidence that plant-based nutrition reduces dietary acid load. J Nutr Sci. 2022;11:e93. 36405093 10.1017/jns.2022.93 PMC9641522

[27] Shivappa N Steck SE Hurley TG Hussey JR Hébert JR. Designing and developing a literature-derived, population-based dietary inflammatory index. Public Health Nutr. 2014;17:1689–96. 23941862 10.1017/S1368980013002115 PMC3925198

[28] Surana S Shah D Gala K. Prevalence of metabolic syndrome in an urban Indian diabetic population using the NCEP ATP III guidelines. J Assoc Physicians India. 2008;56:865–8. 19263684

[29] Molarius A Seidell JC Sans S Tuomilehto J Kuulasmaa K. Educational level, relative body weight, and changes in their association over 10 years: an international perspective from the WHO MONICA Project. Am J Public Health. 2000;90:1260–8. 10937007 10.2105/ajph.90.8.1260 PMC1446346

[30] Hajian-Tilaki K Heidari B. Association of educational level with risk of obesity and abdominal obesity in Iranian adults. J Public Health. 2010;32:202–9. 10.1093/pubmed/fdp083 19689983

[31] Rezazadegan M Mirzaei S Asadi A Akhlaghi M Saneei P. Association between dietary acid load and metabolic health status in overweight and obese adolescents. Sci Rep. 2022;12:10799. 35750714 10.1038/s41598-022-15018-8 PMC9232519

[32] Berkemeyer S. Acid–base balance and weight gain: Are there crucial links via protein and organic acids in understanding obesity? Med Hypotheses. 2009;73:347–56. 19410381 10.1016/j.mehy.2008.09.059

[33] Tangestani H Emamat H Tavakoli A. Association of dietary acid load with metabolic syndrome in overweight and obese women. Int J Vitam Nutr Res. 2022;93:420–6. 35045755 10.1024/0300-9831/a000748

[34] Miyazaki Y Glass L Triplitt C Wajcberg E Mandarino LJ DeFronzo RA. Abdominal fat distribution and peripheral and hepatic insulin resistance in type 2 diabetes mellitus. Am J Physiol Endocrinol Metab. 2002;283:E1135–43. 12424102 10.1152/ajpendo.0327.2001

[35] Wagenknecht LE Langefeld CD Scherzinger AL. Insulin sensitivity, insulin secretion, and abdominal fat: the Insulin Resistance Atherosclerosis Study (IRAS) Family Study. Diabetes. 2003;52:2490–6. 14514631 10.2337/diabetes.52.10.2490

[36] Gui J Li Y Liu H. Obesity-and lipid-related indices as a predictor of obesity metabolic syndrome in a national cohort study. Front Public Health. 2023;11:1073824. 36875382 10.3389/fpubh.2023.1073824 PMC9980350

[37] Mijangos-Trejo A Gómez-Mendoza R Ramos-Ostos MH. Diagnostic accuracy of the Triglyceride–Glucose Index (TyG), TyG body mass index, and TyG waist circumference index for liver steatosis detection. Diagnostics (Basel). 2024;14:762. 38611675 10.3390/diagnostics14070762 PMC11011440

[38] Khamseh ME Malek M Abbasi R Taheri H Lahouti M Alaei-Shahmiri F. Triglyceride glucose index and related parameters (triglyceride glucose-body mass index and triglyceride glucose-waist circumference) identify nonalcoholic fatty liver and liver fibrosis in individuals with overweight/obesity. Metab Syndr Relat Disord. 2021;19:167–73. 33259744 10.1089/met.2020.0109

[39] Li N Tan H Xie A. Value of the triglyceride glucose index combined with body mass index in identifying non-alcoholic fatty liver disease in patients with type 2 diabetes. BMC Endocr Disord. 2022;22:101. 35428219 10.1186/s12902-022-00993-w PMC9011983

[40] Liu L Luo Y Liu M. Triglyceride glucose-related indexes and lipid accumulation products—reliable markers of insulin resistance in the Chinese population. Front Nutr. 2024;11:1373039. 39021592 10.3389/fnut.2024.1373039 PMC11253805

[41] Patel P Abate N. Body fat distribution and insulin resistance. Nutrients. 2013;5:2019–27. 23739143 10.3390/nu5062019 PMC3725490

[42] Soleimani M. Insulin resistance and hypertension: new insights. Kidney Int. 2015;87:497–9. 25723632 10.1038/ki.2014.392

[43] Peng N Kuang M Peng Y. Associations between TyG-BMI and normal-high blood pressure values and hypertension: cross-sectional evidence from a non-diabetic population. Front Cardiovasc Med. 2023;10:1129112. 37168658 10.3389/fcvm.2023.1129112 PMC10164981

[44] Yan J Zhang M-Z He Q-Q. Association of changes and cumulative measures of triglyceride-glucose index-body mass index with hypertension risk: a prospective cohort study. BMC Public Health. 2024;24:2652. 39334211 10.1186/s12889-024-20154-z PMC11438062

[45] Deng D Chen C Wang J Luo S Feng Y. Association between triglyceride glucose-body mass index and hypertension in Chinese adults: a cross-sectional study. J Clin Hypertens (Greenwich). 2023;25:370–9. 36929716 10.1111/jch.14652 PMC10085812

[46] Huang X He J Wu G Peng Z Yang B Ye L. TyG-BMI and hypertension in Normoglycemia subjects in Japan: a cross-sectional study. Diab Vasc Dis Res. 2023;20:14791641231173617. 37209031 10.1177/14791641231173617 PMC10201169

[47] Burger KN Beulens JW Boer JM Spijkerman AM van der A DL. Dietary glycemic load and glycemic index and risk of coronary heart disease and stroke in Dutch men and women: the EPIC-MORGEN study. PLoS One. 2011;6:e25955. 21998729 10.1371/journal.pone.0025955 PMC3187822

[48] Cameron AJ Magliano DJ Shaw JE. The influence of hip circumference on the relationship between abdominal obesity and mortality. Int J Epidemiol. 2012;41:484–94. 22266094 10.1093/ije/dyr198 PMC3324456

[49] Vazquez G Duval S Jacobs DR Jr Silventoinen K. Comparison of body mass index, waist circumference, and waist/hip ratio in predicting incident diabetes: a meta-analysis. Epidemiol Rev. 2007;29:115–28. 17494056 10.1093/epirev/mxm008

[50] Oumer A Ale A Tariku Z Hamza A Abera L Seifu A. Waist-to-hip circumference and waist-to-height ratio could strongly predict glycemic control than body mass index among adult patients with diabetes in Ethiopia: ROC analysis. PLoS One. 2022;17:e0273786. 36350840 10.1371/journal.pone.0273786 PMC9645629

[51] Hare-Bruun H Flint A Heitmann BL. Glycemic index and glycemic load in relation to changes in body weight, body fat distribution, and body composition in adult Danes. Am J Clin Nutr. 2006;84:871–9; quiz 952. 17023715 10.1093/ajcn/84.4.871

[52] Sajjadi SF Milajerdi A Azadbakht L. The association of glycemic index and glycemic load with elevated blood pressure in Iranian women. J Cardiovasc Thorac Res. 2019;11:272–9. 31824608 10.15171/jcvtr.2019.45 PMC6891046

[53] Evans CE Greenwood DC Threapleton DE Gale CP Cleghorn CL Burley VJ. Glycemic index, glycemic load, and blood pressure: a systematic review and meta-analysis of randomized controlled trials. Am J Clin Nutr. 2017;105:1176–90. 28404579 10.3945/ajcn.116.143685

[54] Rouhani MH Kelishadi R Hashemipour M Esmaillzadeh A Azadbakht L. The effect of low glycemic index diet on body weight status and blood pressure in overweight adolescent girls: a randomized clinical trial. Nutr Res Pract. 2013;7:385–92. 24133618 10.4162/nrp.2013.7.5.385 PMC3796664

